# FRCM Confinement of Masonry: Strain Model Assessment and New Proposals

**DOI:** 10.3390/ma17051159

**Published:** 2024-03-01

**Authors:** Annalisa Napoli, Roberto Realfonzo

**Affiliations:** Department of Civil Engineering, University of Salerno, 84084 Fisciano, SA, Italy; rrealfonzo@unisa.it

**Keywords:** fabric-reinforced cementitious matrix (FRCM) systems, external confinement, compressive strain, model assessment

## Abstract

One of the main limitations to the use of fabric-reinforced cementitious matrix (FRCM) composites for the external confinement of masonry is the lack of accurate formulas for estimating the compressive strength and ultimate strain of confined members. With the aim of providing a contribution on the topic, the authors have been carrying out studies on the FRCM-confined masonry for some time and, in a recent study, they proposed some formulations for the prediction of compressive strength. In continuity to that work, an analytical study on the ultimate strain of FRCM-confined masonry is presented in this paper, and preliminary models were derived by considering a wide experimental database compiled from the technical literature. The accuracy of the found relationships was examined based on a comparison with the few formulas published in the literature or reported in international guidelines. To this purpose, it is worth highlighting that the current Italian Guidelines CNR-DT 215/2018 do not provide indications about the estimation of the ultimate strain of FRCM-confined masonry, and the study proposed here attempts to provide a contribution to the mentioned document.

## 1. Introduction and Background of this Study

Fabric-reinforced cementitious matrix (FRCM) composites offer promising solutions for the repair and external strengthening of masonry members. They represent an appealing alternative to fiber-reinforced polymers (FRPs), which experience a decay of performance at high temperatures and inhibit the breathability of the substrate; this latter aspect is a very important requisite to building ancient stone-based masonry buildings.

FRCM composites are also preferred to FRPs from a sustainability perspective, being obtained by embedding an open-grid fabric made of continuous fibers in different types of inorganic matrices (e.g., lime-based mortar, cement-based mortar, and geopolymers). In order to guarantee chemical compatibility with the historical substrate, new lime-based mortars, reinforced with randomly oriented polyvinyl alcohol (PVA) fibers, have lately been under investigation; the random orientation of the PVA fibers, indeed, is also a beneficial effect in seismic areas, since the stress state induced by an earthquake is directionally unknown [1].

The most popular fibers used in FRCM composites are basalt (B), carbon (C), glass (G), and polyparaphenylenebenzobisoxazole (PBO); lately, unidirectional textiles composed of properly spaced ultra-high-tensile strength steel (S) cords are also being used for applications with inorganic matrices, and the resulting composite material is frequently denoted as S-FRCM or, alternatively, steel fiber-reinforced grout (SRG).

The mechanical properties of FRCMs are very sensitive to the type of combination of fibers and matrices and, generally, they are lower than FRPs, due to their lower fiber content or units of matrix volume and lack of full composite action up to failure. Conversely, they are more porous (due to the voids in the inorganic matrix), and therefore, promote the escape and evaporation of moisture from the masonry, thus improving the compatibility between the external reinforcement and the substrate and promoting the permeability of the strengthened surface; also, in strengthening intervention, they assure a reduced invasiveness together with a satisfactory level of reversibility (or at least removability).

Because of the relatively more recent introduction of FRCM composites in the civil engineering field, the number of experimental and theoretical studies available in the literature is lower than the amount of works available for FRPs. A first overview of the research of FRCM strengthening of masonry structures dates from 2018 [2], followed by an updated state of the art published in 2022 [3].

To date, the Italian Guidelines CNR-DT 215 [4], applicable to both concrete and masonry structures, and the American guide ACI 549.6R [5], specifically intended for the repair and strengthening of masonry, represent valuable design tools for practitioners. Despite this, the existing knowledge still requires a great effort by researchers in collecting and analyzing all the available information as well as in validating and/or improving the reliability of the analytical formulations reported in the mentioned design guidelines. Indeed, by accurately examining both guidelines, a rather widespread tendency to adapt the analytical formulations suitable for FRP systems [6,7] to FRCM ones can be noted, sometimes, by readjusting the value assumed by some parameters or, often, leaving the expressions unchanged. From a practical point of view, this way of proceeding can undoubtedly meet the needs of engineers looking for easy-to-apply analytical formulas but, it is equally well known that FRCM systems deserve a more accurate analysis of their performance.

Probably, the noted tendency also lies in the lack of a significant amount of experimental data available at the time of the drafting of the guidelines which is coupled with the considerably higher scatter of results obtained from laboratory tests related to FRCM applications compared to FRP ones. This is the case for the FRCM confinement of masonry of which the first experimental investigations conducted by researchers date back to 2015 [8,9,10] only and, also, the existing knowledge seems to encompass rather uneven and controversial information.

On the other hand, it should also be noted that the interest in deepening the potentiality of FRCM strengthening for masonry structures—and not only for confinement applications—is such that, in the last years, the inorganic matrix has been explored in combination with natural fibers (mainly flax, hemp, or jute) [11,12]. However, the knowledge on these composites, not included in the present study, still requires a deeper understanding.

With the aim of providing a contribution on the topic, the authors have been carrying out studies on FRCM-confined masonry for some time and, in the first stage of our research, they focused on the compressive strength of FRCM-confined masonry. In particular, as a first goal, they published a wide overview of the experimental research available on the topic and proposed preliminary strength models by using a large database of uniaxial compression tests compiled from the literature [13]. With the aim of further advancing the knowledge, an upgrade of the collected database was published in a subsequent paper [14], in which refined relationships for the compressive strength of FRCM-confined masonry were found through best-fit techniques applied to the experimental test results. The suitability of the proposed models was also investigated by dividing the experimental data per the typology of masonry, artificial and natural, with the latter poorly explored in the literature, and further formulations were derived by treating the various types of FRCM systems separately.

In continuity to that work, an analytical study on the axial strain of FRCM-confined concrete is presented in this paper, with the purpose of developing accurate formulations useful for practitioners. In particular, this study focuses on two specific strain parameters identified on the typical axial stress–strain law characterizing the compressive behavior of FRCM-confined masonry, i.e., the strain corresponding to the peak axial stress, namely peak axial strain ($\varepsilon_{mc}$), and the ultimate strain ($\varepsilon_{mcu}$), which is conventionally assumed as the strain corresponding to a fixed strength decay, as better detailed in Section 2.

By using a wide experimental database collecting results in terms of peak and ultimate axial strain, an assessment of the existing strain models published in the literature or reported in international guidelines was first performed. Then, with the aim to provide a contribution in terms of updating/integrating the mentioned international guidelines [4,5], the most accurate formulas were developed by applying error minimization techniques to the experimental test results. Concerning this, it is worth highlighting that the Italian Guidelines CNR-DT 215/2018 [4] do not provide indications about the estimation of peak/ultimate strain of FRCM-confined masonry, and the study proposed here can contribute to filling this gap.

## 2. Structural Behavior of FRCM-Confined Masonry

It is well known that the stress–strain behavior of FRCM-confined masonry subjected to uniaxial compression is different from that experienced in the case of FRP confinement [15,16,17]. Generally, for a given type of fabric, embedment with an inorganic matrix makes masonry confinement less effective than epoxy-based application and significantly dependent on cracking development in the mortar. Indeed, in the case of FRP confinement, full composite action can be assumed between the fiber and the epoxy-based matrix up to failure, and external jacket rupture is sudden, brittle, and caused by the achievement of ultimate fiber strain. Conversely, in FRCM confinement, full composite action is assured up to mortar cracking after which the fabric tends to slip within the matrix and the constituent yards start to be loaded differently, thus causing an even greater shift of one yard concerning the other. As a result, the failure of the confined member is typically less sudden, less brittle, and can be classified by three different modes: jacket failure (JF), debonding of the external reinforcement (DB), and fiber–matrix slippage (S). However, the JF and DB modes cannot occur without the preliminary occurrence of significant fiber–matrix slippage, and the JF mode is not comparable to the jacket rupture observed for FRP confinement. It is more related to a non-uniformly distributed tensile load between the yards, which leads some of them to achieve rupture before the others due to excessive stress [3,17,18].

**Figure 1:**
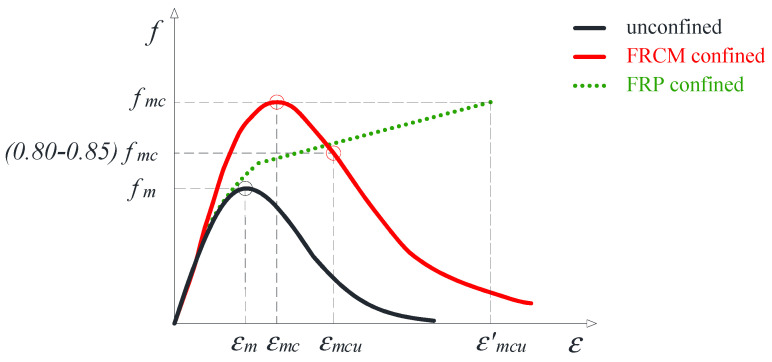
Typical stress–strain behavior of masonry confined with FRP/FRCM under compression.

Figure 1 shows the ideal axial stress–strain (*f*–ε) law of a masonry column confined by an FRCM (red curve in Figure 1) under uniaxial compression, which is compared with the typical response observed in the case of FRP confinement (dotted green curve in Figure 1) and with unconfined masonry behavior (black curve in Figure 1).

The stress and strain parameters indicated in Figure 1 have the following meanings:

- For unconfined masonry, $f_{m}$ is the compressive strength and $\varepsilon_{m}$, the corresponding strain.
- For FRP-confined masonry, $f_{mc}$ is the compressive strength and ${\varepsilon \prime }_{mcu}$, the corresponding strain that, in the case of an ascending stress–strain response, coincides with the ultimate one (i.e., the strain attained at jacket failure).
- For FRCM-confined masonry, $f_{mc}$ is, again, the compressive strength while $\varepsilon_{mc}$ is the corresponding strain; $\varepsilon_{mcu}$ is the conventional ultimate strain that, according to [19], is assumed to be in the range 80–85% of the peak axial stress, identified at the post-peak branch since, at that stage, the member’s structural capacity is believed to be rather compromised.

It is worth highlighting that, in the case of the FRP confinement, the ascending stress–strain law is typically observed as long as the effective lateral confining pressure exerted by the external jacket, normalized to the compressive strength of the unconfined member, is higher than 5–8%. This is the threshold generally considered to define an FRP confining system as “effective” [4,5,20].

In the case of FRCM confinement, instead, the presence of the mortar cracking which affects matrix-fiber interaction is generally responsible for post-peak softening behavior. In particular, if the level of bonding at the fiber–matrix interface is very low, stress may be suddenly transferred to the fibers which, subjected to uneven tensile stresses, can lead to a premature failure of the FRCM system at a level of load significantly lower than the mesh tensile strength.

Of course, the stress–strain with a post-peak softening branch typically observed in FRCM-confined members can also be experienced by FRP-confined members, but typically when the FRP confining system is considered “not effective” (i.e., normalized confining pressure lower than 5–8%). In this case, by neglecting the effect of the inorganic matrix, the models available for FRP confinement might be adapted to FRCM confinement.

Conversely, for FRCM-confined members, the stress–strain response like for FRP confinement might be theoretically experienced in the presence of a high-performance inorganic matrix coupled with a good matrix bond; if both conditions exist, an ascending stress–strain curve might be obtained since the stresses are gradually transferred to the fiber mesh while the crack phenomenon evolves up to the complete damage of the mortar. However, based on the current knowledge on the topic and, mainly, the high scatter of test results available in the literature, it is very hard to establish threshold values based on an ascending stress–strain response rather than on a post-peak softening response, which is expected. Furthermore, it is worth highlighting that the installation procedure of the external reinforcement plays a key role in FRCM confinement efficacy, since a wet lay-up application may affect the perfect alignment and uniform arrangement of fiber filaments within each fiber bundle. As a result, under applied load, the stress distribution inside the fiber bundles can be non-uniform, and debonding at the fiber–matrix interface can be experienced [17].

Based on the above considerations, the development of accurate analytical models for predicting the compressive strength and the peak and/or ultimate axial strain of FRCM-confined masonry is an issue since these models should account for all the mentioned physical and behavioral factors, and their greater or lesser relevance relies on the specific FRCM system employed for column confinement. However, it is equally true that, from an engineering perspective, there is a demand for the development of easy-to-apply formulas that, beyond the difficulties arising from the theoretical interpretation of the “matrix-effect”, are capable of roughly predicting the structural performance of FRCM-confined masonry. To this purpose, the study presented in this paper aims to propose simplified formulas for the estimation of both the peak axial strain $\varepsilon_{mc}$ and the ultimate strain $\varepsilon_{mcu}$ corresponding to 80–85% strength decay (see Figure 1).

## 3. Overview of the Existing Strain Models

**Figure 2:**
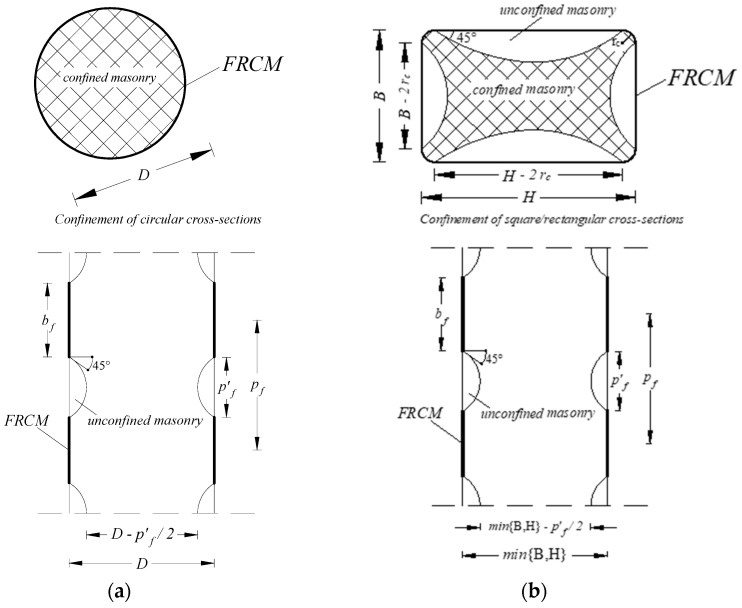
Schematic representation of FRCM-confined masonry columns in the presence of discontinuous wrap configuration: (**a**) columns with a circular section (CS); (**b**) columns with a square/rectangular section (RS).

Table 1 shows the few analytical formulas found in the literature [18,21,22] and in the American guide ACI 549.6R [5], which are specified for both circular sections (CS) and square/rectangular sections (RS) in the presence of full wrapping (F-W) or discontinuous wrapping (DIS-W). For the meanings of most of the symbols reported in Table 1, reference to Figure 2 can be made; the remaining ones are detailed in the following.

The considered formulas divide into two groups: (a) formulas for the prediction of the peak axial strain $\varepsilon_{mc}$ (Table 1a) and (b) formulas for the prediction of the ultimate strain $\varepsilon_{mcu}$ (Table 1b).

In all the models, the estimation of the peak/ultimate strain relies on the parameter ${\bar{f}}_{l,eff}$ which indicates the effective lateral confining pressure exerted by the FRCM confining system ($f_{l,eff}$) normalized to the compressive strength of the unconfined masonry $f_{m}$. In turn, $f_{l,eff}$ is expressed by the following:

(1) $$f_{l,eff}=k_{eff}\cdot f_{l}$$

In Equation (1)

- $k_{eff}$ = $k_{H}\cdot k_{V}$ is the confinement efficiency factor, with $k_{H}$ (≤1) and $k_{V}$ (≤1) being the horizontal and vertical confinement efficiency factors, respectively, estimated by the various models.
- $f_{l}$ is the lateral confining pressure given by
  (2) $$f_{l}=\frac{1}{2}\cdot \rho_{f}\cdot E_{f}\cdot \left(k_{\varepsilon }\cdot \varepsilon_{f,u}\right)$$
  where
- $\rho_{f}$ is the geometric strengthening ratio related to the FRCM system, which is dependent on both the equivalent thickness of the single FRCM layer ($t_{f,j}$) multiplied by the number of FRCM layers ($n_{f}$), i.e., $t_{f}=t_{f,j}\cdot n_{f}$, and the cross-section geometry of the confined member;
- $E_{f}$ and $\varepsilon_{f,u}$ are the elastic modulus and the ultimate tensile strain of the dry strengthening sheet, respectively;
- $k_{\varepsilon }$(≤1) is the strain efficiency factor defined as the ratio between the ultimate hoop strain experimentally measured in the FRCM jacket (*ε_j,u_*) and the ultimate strain found from fiber coupon tensile tests (*ε_f,u_*).

By focusing on Table 1a, it can be noted that the linear and non-linear models proposed by Krevaikas [22], namely *Model 1A* and *1B*, show the same structure of the relationship proposed by Koutas and Bournas [21], namely *Model 2*, except for the exponent “*n*”, whose value changes with FRCM confinement. Basically, the structure of these models is that typically adopted in the case of FRP confinement [6,7], meaning that the influence of the inorganic matrix on strain prediction is neglected.

Conversely, the model by Micelli et al. [18], namely *Model 1D*, and the ACI model proposed by the RILEM Committee TC 250-CSM [5], namely *Model 1E*, take into account the contribution of the inorganic matrix through the parameters $k_{mat\;}$ and $k^{\prime }$, respectively; the mathematical expression for the estimation of both parameters is the same except for the different value suggested for the exponent of the term $\left(\rho_{mat}\cdot \frac{f_{mat,c}}{f_{m}}\right)$. Basically, the matrix effect is made to depend on (a) the matrix reinforcement ratio $\rho_{mat}$ in which $t_{mat}$ denotes the total thickness of the inorganic matrix employed in the FRCM jacket and (b) the ratio between the compressive strength of the inorganic matrix $\left(f_{mat,c}\right)$ and the compressive strength of the unconfined masonry $\left(f_{m}\right)$.

Except for the ACI model, all the formulas reported in Table 1a (but also for the model by Micelli et al. [18] in Table 1b) were originally developed to be applied to FRCM-confined masonry columns in the F-W configuration due to a lack of experimental data concerning discontinuous wrapping. Therefore, the expression reported in Table 1a for the estimation of the vertical confinement efficiency’s coefficient $k_{V}$ is that reported in American guide ACI 549.6R [5] which, in turn, is the same already proposed for FRP confinement applications [6].

Concerning Table 1b, it is worth highlighting that the prediction of the ultimate strain is not easy due to the lack of a significant amount of experimental data useful for model calibration. Additionally, it is very important to establish what is meant by ultimate deformation and what the stress–strain response assumed for its prediction is. For instance, the two models reported in Table 1b are based on a different definition of the ultimate strain which is the result of a different stress–strain response assumed for FRCM-confined masonry. In particular, the strain model proposed by the ACI 549-L Committee [5] kept the same structure already proposed by the American guide ACI 440.2R for FRP applications [7] and assumed a hardening post-peak stress–strain behavior similar to that shown by the dotted green curve in Figure 1. In this case, the ultimate axial strain coincides with the strain at peak stress $f_{cm}$ and corresponds to ${\varepsilon^{\prime }}_{mcu}$ in Figure 1. Conversely, the model by Micelli et al. [18] was developed by considering a more realistic stress–strain behavior experimentally observed for FRCM-confined members (see the red curve in Figure 1) based on which the ultimate strain represents the strain attained at 80–85% of the axial strength on the softening post-peak branch. According to the authors, at 15–20% strength decay, the column is significantly compromised and unable to carry new or residual loads. Also, the model does not include the contribution of the FRCM matrix, since at that stage, the matrix is assumed to be significantly damaged.

## 4. Collection of Experimental Datasets

Table A1, Table A2, Table A3, Table A4 and Table A5 in Appendix A report all the information related to the experimental datasets collected in the database [8,9,10,21,23,24,25,26,27,28,29,30,31,32,33,34,35,36]. Each dataset was identified by following the “Design by testing” approach recommended by Annex D of Eurocode 0 [37]. It is representative of a group of N experimental tests performed by the same researchers and characterized by uniformity in terms of the following:

- material and masonry arrangement;
- specimen size;
- geometry, typology of the FRCM system, and confinement layout;
- mechanical properties of the fiber mesh;
- thickness and mechanical properties of the inorganic matrix employed in the FRCM system;
- compressive strength of the unconfined masonry.

The experimental result attributed to each dataset is provided in Table A1 in terms of the compressive strength of the FRCM-confined masonry normalized with respect to the strength of the unconfined masonry ($\frac{f_{mc}}{f_{m}}$), as well as the axial strain at the peak stress and conventional ultimate strain, both normalized to the axial strain at the peak stress of the unconfined masonry, i.e., $\frac{\varepsilon_{mc}}{\varepsilon_{m}}$ and $\frac{\varepsilon_{mcu}}{\varepsilon_{m}}$. Each result represents the average value calculated from the collected N tests; for more details, reference to [38] can be made.

Before organizing the database into datasets, great care was taken in collecting the information, especially for the results related to the conventional ultimate strain $\varepsilon_{mcu}$. In particular, this value was either directly found in the scientific paper (when specified that it was referring to 15–20% of the strength decay on the post-peak branch) or accurately derived by the authors on the stress–strain laws when published in the papers; in this last case, the conventional ultimate strain was always taken at 20% of the strength decay.

Table 2 summarizes the number *n* of datasets and the corresponding number *N* of specimens to which they refer, distributed per FRCM system. By focusing on the peak strain, C-, G-, and S-FRCM are the systems with the greater number of datasets; conversely, only 10 datasets were found for the PBO-FRCM system for which results in terms of ultimate strain were not available.

Table 3, instead, provides the distribution of datasets per masonry type within each FRCM system. In particular, most of compression tests gathered in the database were performed on specimens made of clay brick masonry (CB), which, in terms of datasets available for the parameter $\varepsilon_{mc}$, represent 79% of the database; the few datasets related to natural stone masonry (NS) mainly concerned limestone blocks (LS) and only a few datasets belonging to S- and G- FRCM systems included samples made by tuff units (TUs).

**Figure 3:**
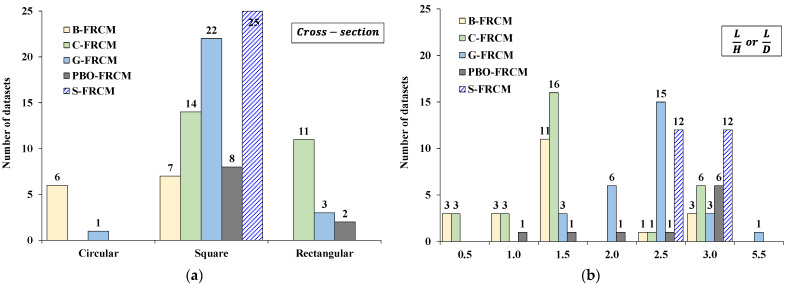
Distributions of datasets based on the (**a**) shape of the column cross-section; (**b**) aspect ratio L/H or L/D.

Figure 3 show the distribution of all datasets collected in the database based on the shape of the column’s cross-section (circular—C, square—S, and rectangular—R), Figure 3a, and the aspect ratio L/H (for S/R specimens) or L/D (for C specimens), Figure 3b, with L being the height of the member and H or D, the longer side or the diameter of the cross-section, respectively.

As noted, most datasets included square specimens with side dimensions mostly concentrated about the value of 250 mm, while very few datasets concerned cylindrical members with a diameter D between 80 and 95 mm (only available for B- and G- specimens). Regarding the prismatic specimens with a rectangular cross-section, the side ratio H/B ranged between 1.5 and 2.5 (the most investigated value was 2, with B being the shorter side of the cross-section). In terms of the aspect ratio, instead, the most used values were 1.5, 2.5, and 3, with less scattering of data in the case of the S-FRCM system.

Additional information on the collected database can be obtained by analyzing the datasets reported in Table A1 or in a recently published paper focusing on the prediction of the compressive strength of FRCM-confined concrete [14].

## 5. Assessment of the Existing Strain Models

The accuracy of models presented in Section 2 was examined based on the available experimental database. For each model, the model performance was first qualitatively analyzed in terms of comparison between the theoretical prediction of the peak or ultimate strain of the *i*-th dataset of the database and the corresponding experimental value; this comparison allows for drawing indications on the conservativeness or non-conservativeness of the estimates obtained from the different models.

Furthermore, a more quantitative analysis of the various models was performed in terms of distribution of the experimental-to-theoretical ratios $\delta_{i}$ expressed by

(3) $$\delta_{i}=\frac{\varepsilon_{mc,i}^{exp}}{\varepsilon_{mc,i}^{th}}\;\mathrm{peak}\mathrm{strain},\;\delta_{i}=\frac{\varepsilon_{mcu,i}^{exp}}{\varepsilon_{mcu,i}^{th}}\;\mathrm{ultimate}\mathrm{strain}$$

where

- the subscript “*i*” stands for the *i*-th datasets;
- $\varepsilon_{mc,i}^{exp}$ and $\varepsilon_{mcu,i}^{exp}$ are the *i*-th experimental values of the axial strain at the peak and ultimate, respectively, and $\varepsilon_{mc,i}^{th}$ and $\varepsilon_{mcu,i}^{th}$ are the corresponding theoretical values estimated according to the models.

In detail, the values of the median ${∆}_{\delta }$, mean $\delta_{m}$, standard deviation $\sigma_{\delta }$, and asymmetry $\gamma_{\delta }$ of the distribution were examined, together with the total error $\delta_{n}$, which is expressed by

(4) $$\delta_{n}=\sum_{i=1}^{n}{\left(\varepsilon_{mc,i}^{exp}-\varepsilon_{mc,i}^{th}\right)}^{2}\;\mathrm{peak}\mathrm{strain},\;\delta_{n}=\sum_{i=1}^{n}{\left(\varepsilon_{mcu,i}^{exp}-\varepsilon_{mcu,i}^{th}\right)}^{2}\;\mathrm{ultimate}\mathrm{strain}$$

where *n* is the number of datasets considered in the analysis.

Finally, a possible measure of the error in the model prediction could be obtained by considering the mean absolute percentage error (MAPE), which is expressed by

(5) $${\left(E_{rr}\right)}_{m}=\frac{\sum_{i=1}^{n}\left|E_{i}\right|}{n}$$

where $E_{i}$ is the *i*-th error between the experimental and model prediction, given by

(6) $$E_{i}=\frac{\left(\varepsilon_{mc,i}^{exp}-\varepsilon_{mc,i}^{th}\right)}{\varepsilon_{mc,i}^{exp}}\cdot 100\;\mathrm{peak}\mathrm{strain},\;E_{i}=\frac{\left(\varepsilon_{mcu,i}^{exp}-\varepsilon_{mcu,i}^{th}\right)}{\varepsilon_{mcu,i}^{exp}}\cdot 100\;\mathrm{ultimate}\mathrm{strain}$$

### 5.1. Prediction of the Peak Axial Strain

Table 4 reports the results of the statistical analysis performed on the model error $\delta_{i}$, calculated according to Equation (3). In particular, Table 4a reports the results of the analysis obtained by applying *Model 1A* to *1C*, while Table 4b focuses on the remaining *Model 1D* and *1E*; such analyses were performed by either considering all datasets together or treating the FRCM systems separately.

**Figure 4:**
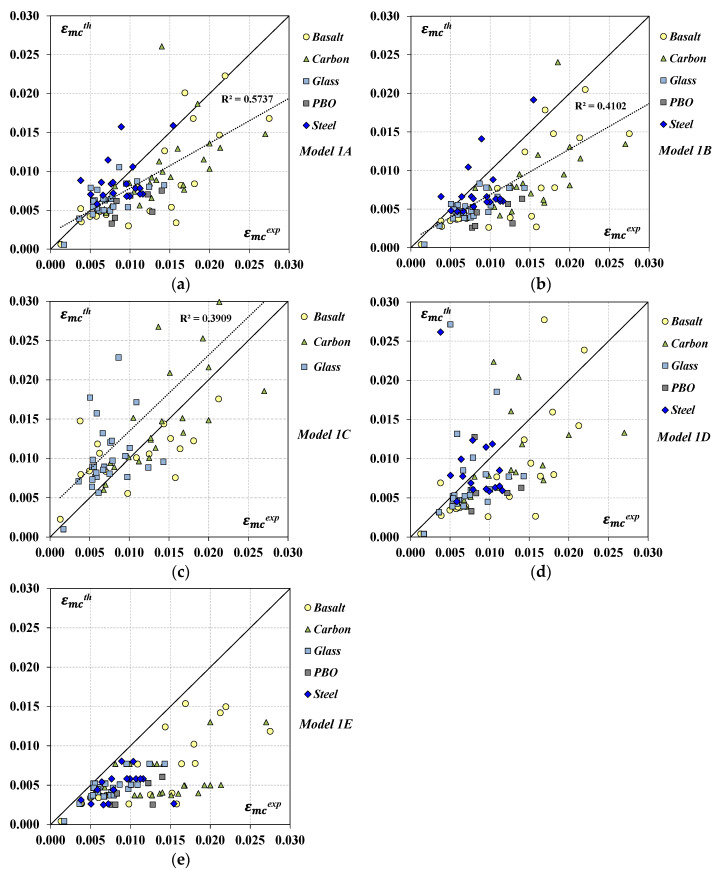
Predicted vs. experimental values in terms of peak strain: (**a**) *Model 1A*; (**b**) *Model 1B;* (**c**) *Model 1C*; (**d**) *Model 1D*; (**e**) *Model 1E*.

Furthermore, the accuracy of the models’ predictions was also qualitatively examined from Figure 4, where the theoretical values in terms of peak strain $\varepsilon_{mc}^{th}$ were plotted and compared to the experimental results. The bisector corresponds to perfect agreement between predictions and tests; therefore, points falling in the lower part of the graph indicate conservative predictions whereas points falling over the line are representative of non-conservative situations.

**Figure 5:**
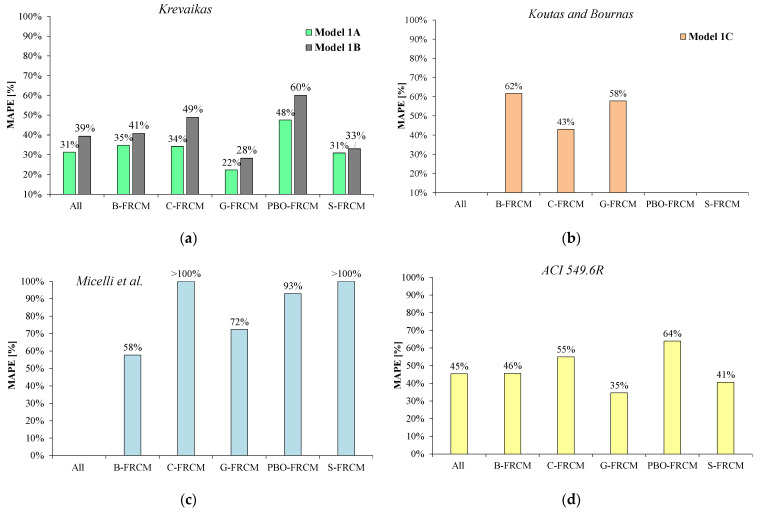
Model errors in terms of MAPE in the estimation of the peak strain: (**a**) model by Krevaikas [22]; (**b**) model by Koutas and Bournas [21]; (**c**) model by Micelli et al. [18]; (**d**) model by ACI549.6R [5].

Finally, the bar charts in Figure 5 show the model errors estimated according to the MAPE (Equation (5)) by both considering all datasets together and dividing them per FRCM system. In particular, the bar chart in Figure 5a focuses on the predictions obtained from *Model 1A* and *1B*, while Figure 5b,c,d are related to *Model 1C*, *1D*, and *1E*, respectively.

It is worth highlighting that the analysis was performed on a slightly reduced number *n* of datasets with respect to those indicated in Table 2 and reported in the general database (Table A1). Specifically, some datasets were not considered in the analysis since the associated experimental results in terms of $\varepsilon_{mc}$ resulted in being approximately equal or even lower than the corresponding values obtained for unconfined members (i.e., $\varepsilon_{mc}\leq \varepsilon_{m}$), thus making the FRCM confinement ineffective. Other (few) datasets, instead, were neglected because the experimentally observed behavior was significantly different from that shown in Figure 1 (for instance, stress–strain laws showing double stress peaks, deserving more investigation, were noted in some cases). Therefore, Table 4a,b report the number of datasets effectively considered in the analysis per each FRCM system; in comparison with the number of datasets provided in Table 2, it was noted that, of the eleven removed datasets, five belonged to S-FRCM, four, to PBO-FRCM, one, to C-FRCM, and one, to G-FRCM. By focusing on Table 4a and on the related Figure 4a–c and Figure 5a,b, it can be noted that the linear model by Krevaikas [22] showed a generally better performance than the non-linear *Model 1B* for any type of FRCM system. The total error $\delta_{n}$ was systematically lower in the case of *Model 1A* and the standard deviation $\sigma_{\delta }$ as well, even though the latter was quite high for all the FRCM systems and, mainly, in the case of the B fibers. As shown in Figure 4a,b, both models mostly provided conservative predictions and the linear correlation coefficient R^2^ was lower than 0.6; in terms of MAPE errors, Figure 5a highlights the best performance of the linear *Model 1A* with respect to *Model 1B* with an overall MAPE error of 31% calculated on all datasets against 39% found by applying *Model 1B*.

Also, both models provided less accurate predictions in the case of the PBO-FRCM system and their application yielded estimates that were too conservative.

Conversely, *Model 1C* often provided non-conservative predictions as shown in Figure 4c and by the parameter $\delta_{m}$ in Table 4a. Despite a higher total error $\delta_{n}$, the standard deviations $\sigma_{\delta }$ were always lower than the values found, for a given FRCM, by the application of *Model 1A* and *1B*; however, concerning the MAPE analysis, the model did not provide a satisfying result since the errors were over 43%.

By focusing on Table 4b and the related Figure 4d,e and Figure 5c,d, it can be noted that, based on the experimental database available so far, the models accounting for the contribution of inorganic matrix showed some criticisms, which need to be addressed. Indeed, the results of the statistical analysis in Table 4b are not very encouraging especially for *Model 1D*, where the values obtained for the standard deviation $\sigma_{\delta }$ and the total error $\delta_{n}$ were significantly higher. The main concerns regard the estimation of the peak strain in the case of C-FRCM and S-FRCM systems for which the model accuracy was questionable for the significant scatter between the predicted and experimental values (too high $\delta_{n}$ values); this was confirmed by the MAPE values shown in Figure 5c which, for these two FRCM systems, were greater than 100%. By looking at the structure of this formula (see Table 1a), the authors believe that model prediction is too dependent on the value assumed by the parameter $k_{mat}$ which linearly multiplies the lateral confining pressure.

**Figure 6:**
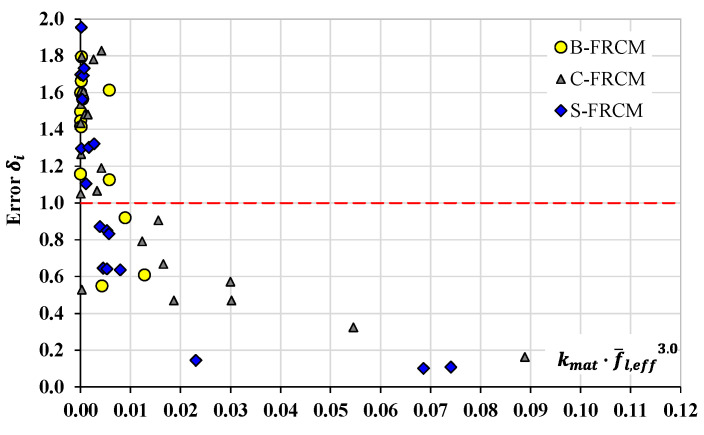
Relationship between errors $\delta_{i}$ and ($k_{mat}\cdot {{\bar{f}}_{l,eff}}^{3}$): model by Micelli et al. [18].

For a better understanding of the suitability of *Model 1D*, Figure 6 shows the relationship between the product ${k_{mat}\cdot {\bar{f}}_{l,eff}}^{3.0}$ and the corresponding error $\delta_{i}$ with reference to the experimental datasets belonging to the B-, C-, and S-FRCM systems; as observed in Figure 5c, this model provided the lowest MAPE for the B-FRCM system (even though it was rather high) and the highest values for both the C- and S-FRCM systems (errors of over 100%).

It can be noted that, for values of the product ${k_{mat}\cdot {\bar{f}}_{l,eff}}^{3.0}$ lower than 0.01, the model mostly seemed to be excessively conservative, with $\delta_{i}$ values significantly greater than the unit. Conversely, by increasing the value of the product ${k_{mat}\cdot {\bar{f}}_{l,eff}}^{3.0}$ over 0.01, the model became even more significantly non-conservative, maybe implying that, the use of a high-performing inorganic matrix (high value of $k_{mat}$) or a high value of the lateral confining pressure ${\bar{f}}_{l,eff}$ does not produce a significant increase in the peak axial strain. Therefore, as our preliminary consideration, the mentioned product should be limited to 0.01. Also, as shown, the better model performance for the B-FRCM system seems to have only depended on the available datasets which always yielded ${k_{mat}\cdot {\bar{f}}_{l,eff}}^{3.0}\leq \;1$ (only one dataset had a value slightly greater than 1) and, therefore, they did not induce the model to completely fail the prediction.

### 5.2. Prediction of the Ultimate Axial Strain

Table 5 reports the results of the statistical analysis performed on the model error $\delta_{i}$ calculated according to Equation (3). It is worth mentioning that only *Model 2B* was considered here since the datasets considered in the analyses referred to compression tests showing stress–strain responses with a softening branch. As a result, *Model 2A* is believed to be unsuitable for the available datasets.

Like the study of the peak axial strain, the results in Table 5 were obtained by either considering all datasets together or treating the FRCM systems separately. It is worth highlighting that the PBO-FRCM system was not included in the analysis due to the lack, to date, of experimental datasets providing results in terms of ultimate strain.

**Figure 7:**
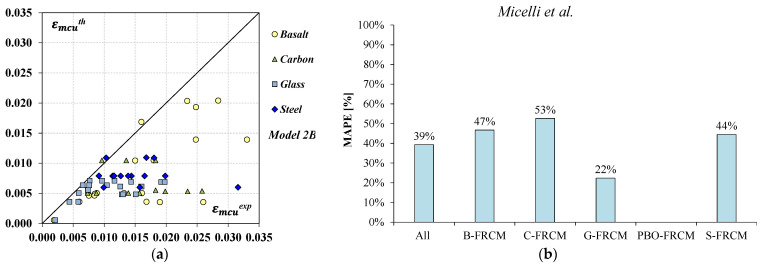
Estimates of the ultimate strain: (**a**) predicted vs. experimental values; (**b**) model error in terms of MAPE according to the model by Micelli et al. [18].

Furthermore, the accuracy of the model’s predictions was qualitatively examined from Figure 7a where the theoretical values in terms of ultimate strain $\varepsilon_{mcu}^{th}$ were plotted and compared to the experimental results. Finally, the bar chart in Figure 7b shows the model errors estimated according to the MAPE (Equation (5)) by both considering all datasets together and dividing them by their corresponding FRCM system.

The results in Table 5 and Figure 7 highlight that the model by Micelli et al. [18] provided too many conservative estimates for all the FRCM systems; the points plotted in Figure 7a are mostly distributed below the bisector, while the mean value of the model errors δ was slightly lower than 2, only for the G-FRCM system, and the standard deviation $\sigma_{\delta }$ was significantly high. By looking at Figure 7b, it can be noted that the MAPE values, except for the G-FRCM system, were higher than 40%.

Overall, the obtained results emphasize the need to recalibrate the coefficients characterizing the considered model to improve its prediction accuracy.

## 6. New Proposals

Based on the assessment of the existing strain models, new proposals for the estimation of the peak and ultimate strain of FRCM-confined masonry were derived through best-fit analyses applied to the available experimental datasets.

Concerning the prediction of the peak strain $\varepsilon_{mc}$, a general two-parameter formula described by Equation (7) was considered which was derived from the relationships proposed by Krevaikas [22], i.e.,

(7) $$\varepsilon_{mc}=\varepsilon_{m}+\alpha_{1}\cdot {{\bar{f}}_{l,eff}}^{\alpha_{2}}$$

where $\alpha_{1}$ and $\alpha_{2}$ are the finetuning parameters to be calibrated through a proper error minimization technique.

Concerning the prediction of the ultimate strain $\varepsilon_{mcu}$, due to the poor availability of formulas, the model by Micelli et al. [18] was again considered and rewritten as the following more general three-parameter expression:

(8) $$\varepsilon_{mcu}=\varepsilon_{m}\left[1.36+\alpha_{1}\cdot {\left(\frac{\varepsilon_{f,u}}{\varepsilon_{m}}\right)}^{\alpha_{3}}\cdot {{\bar{f}}_{l,eff}}^{\alpha_{2}}\right]$$

where $\alpha_{1}$, $\alpha_{2}$, and $\alpha_{3}$ are, again, the finetuning parameters to be calibrated through a proper error minimization technique.

To this purpose, the already mentioned MAPE, expressed by Equations (5) and (6), was selected to minimize the error between experiments and predictions. Based on authors’ experience, the MAPE technique yields the most conservative results in terms of model calibration [14,24].

However, it is highlighted that the proposed values of $\alpha_{i}$ parameters are those that, at the same time, provide the lowest MAPE and lead to an overall improvement in the values of the parameters included in the statistical analysis. The results of the calibration procedures applied to the estimation of both the peak and ultimate strain are detailed in the following sections.

### 6.1. Proposal for the Prediction of the Peak Axial Strain

Table 6 and Table 7 report the results of the best-fit analyses performed on different groups of considered experimental datasets. In particular, Table 6 provides the results of the calibration procedure applied to all 101 datasets in terms of ${\left(E_{rr}\right)}_{m}$ values, with the purpose of finding a relationship suitable for any FRCM system; this relationship, labelled *Proposal 1*, was characterized by $\alpha_{1}=0.006$ and $\alpha_{1}=0.37$. The results of the statistical analysis were, then, applied to all datasets and specifically, datasets belonging to each FRCM system, to also investigate the accuracy of the proposed model in the various cases.

With the purpose of finding ad hoc formulation for the specific confining system, Table 7 provides the results of the calibration procedure and of the corresponding statistical analysis applied separately to datasets belonging to each FRCM system; the best-fit relationships found are labelled as *Proposal 2*.

**Figure 8:**
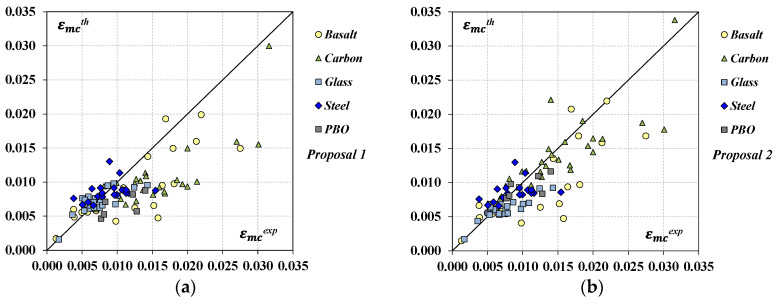
Predicted vs. experimental values in terms of peak strain: (**a**) *Proposal 1*; (**b**) *Proposal 2*.

As expected, the new formulas provide an overall improvement in the strain prediction for all the FRCM systems and, therefore, a better agreement with the experimental results as shown in Figure 8, where the experimental datasets in terms of ${\varepsilon_{mc}}^{exp}$ were plotted together with their corresponding ${\varepsilon_{mc}}^{th}$ values. The plot in Figure 8a is related to the predictions obtained by applying *Proposal 1* while that in Figure 8b, to the estimates by *Proposal 2*; a higher concentration of data about the bisector can be noted.

**Figure 9:**
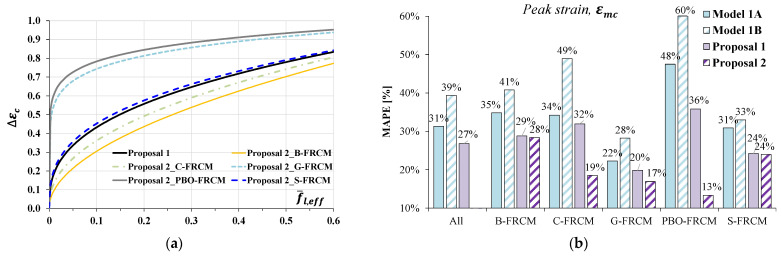
Estimates of the peak strain: (**a**) relationship between ${∆\varepsilon }_{c}$ and ${\bar{f}}_{l,eff}$; (**b**) model error in terms of MAPE according to the *Model 1A* [22], *Model 1B* [22], *Proposal 1* and *Proposal 2*.

In Figure 9a, the strain increase ${∆\varepsilon }_{c}=\varepsilon_{mc}-\varepsilon_{m}$ estimated by applying the best-fit models was plotted as a function of the effective confinement pressure ${\bar{f}}_{l,eff}$. It can be observed that *Proposal 1* is very similar to *Proposal 2* found for the S-FRCM system and rather different from those found for the G- and PBO- FRCM systems; for a given lateral confining pressure, these two FRCM systems seem to provide a significantly greater strain increase with respect to the counterpart composites.

Figure 9b shows a comparison in terms of MAPE values between the original models proposed by Krevaikas [22] (*Model 1A* and *Model 1B)* and the new relationships found through best-fit analyses. The bar chart confirms the better accuracy of the proposed models, with significant improvements especially in the cases of the PBO-, G-, and C- systems for which the MAPE values went down to 20% when using *Proposal 2.*

### 6.2. Proposal for the Prediction of the Ultimate Axial Strain

Similarly to what was performed for the prediction of the peak strain, Table 8 and Table 9 report the results of the best-fit analyses performed on the different groups of considered experimental datasets. Specifically, Table 8 provides the results of the calibration procedure applied to the available 68 datasets in terms of ${\left(E_{rr}\right)}_{m}$ values, with the purpose of finding a relationship suitable for any FRCM system; this relationship, labelled *Proposal 1*, was characterized by $\alpha_{1}=0.64$, $\alpha_{2}=0.53$, and $\alpha_{3}=1.00$. The results of the statistical analysis were then applied to all datasets and datasets specifically belonging to each FRCM system to also investigate the accuracy of the proposed model in the various cases.

With the purpose of finding the ad hoc formulation for the specific confining system, Table 9 provides the results of the calibration procedure and of the corresponding statistical analysis applied separately to datasets belonging to each FRCM system; the best-fit relationships found are labelled as *Proposal 2*.

**Figure 10:**
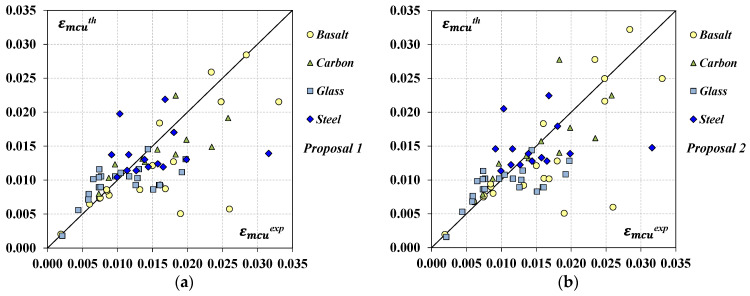
Predicted vs. experimental values in terms of ultimate strain: (**a**) *Proposal 1*; (**b**) *Proposal 2*.

As expected, the new formulas provide an overall improvement in the strain prediction for all the FRCM systems with respect to the original model by Micelli et al. [18] (*Model 2B*); a significant reduction in the total error $\delta_{n}$ was obtained and, also, a better agreement with the experimental results can be noted from Figure 10, where the experimental datasets in terms of ${\varepsilon_{mc}}^{exp}$ were plotted together with their corresponding ${\varepsilon_{mc}}^{th}$ values. The plot in Figure 10a is related to the predictions obtained by applying *Proposal 1* while that in Figure 10b, to the estimates by *Proposal 2*.

**Figure 11:**
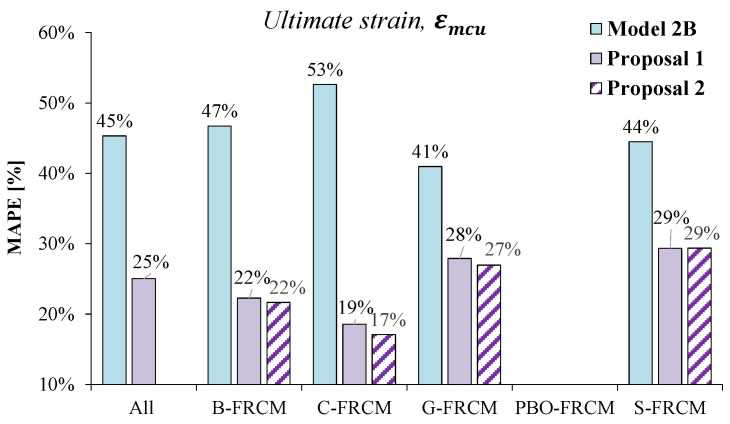
Estimates of the ultimate strain: model error in terms of MAPE according to *Model 2B* [18], *Proposal 1*, and *Proposal 2*.

Finally, the plot in Figure 11 shows the comparison in terms of MAPE values between the original model proposed by Micelli et al. [18] (*Model 2B)* and the new relationships found through best-fit analyses. The bar chart confirms the better accuracy of the proposed models, with significant improvements especially in the case of the C-FRCM. Indeed, the model errors went down below the 30% threshold and, also, it was proven that *Proposal 1* could be successfully used for all the FRCM systems, since the MAPE values calculated for both *Proposal 1* and *Proposal 2* were very similar.

## 7. Conclusions

In this paper, a first analytical study on the compressive strain of masonry members confined with FRCM systems has been presented. This study was based on a wide experimental database compiled from the literature which included the results of 243 compression tests on masonry members confined by FRCM, most of which with had square or rectangular cross-sections and were made by properly assembling clay bricks.

The collected database was a valuable tool to check the reliability of existing formulas in the estimation of the axial strain at peak strength, $\varepsilon_{mc}$, and of the ultimate strain, $\varepsilon_{mcu}$; the latter was conventionally assumed to be at 15–20% of the strength decay on the softening branch of the stress–strain law.

Despite the well-established belief of the influence of an inorganic matrix on the compressive behavior of FRCM-confined masonry, the assessment procedure highlighted that the existing formulas for peak strain prediction that account for an inorganic matrix are less effective than simplified models very similar to those used for FRP confinement.

This result is motivated by the high scatter of data related to the mechanical properties of the inorganic matrix collected in the database which affected the performed analyses. As a result, the models did not account for the contribution of inorganic matrix, and particularly, those proposed by Krevaikas showed a better agreement with the experimental results mainly for G- and S-FRCM systems.

Regarding the ultimate strain, instead, the study of the literature further highlighted that, except for very few cases, FRCM-confined specimens experimentally experienced stress–strain behaviors with softening branches and therefore, were not comparable to those typically exhibited by FRP-confined specimens. This is the reason for which the relationship currently included in the American guide ACI 549.6R/2020—following the “ACI approach”—is believed to not be applicable to the collected database.

Based on the results of our model assessment, new proposals were developed by performing best fitting of the experimental datasets and calibrating the key parameters of the selected models for the prediction of the peak and ultimate strain of FRCM-confined masonry. Specifically, for both strain parameters, we proposed the following formulas: *Proposal 1*, suitable for any FRCM system, and *Proposal 2*, developed ad hoc for each FRCM system.

As expected, the new formulas improved the peak strain prediction for all the FRCM systems by providing a better agreement with the experimental results. Mainly in the case of the PBO-, G-, and C- systems, the values of the mean absolute percentage error (MAPE) between the experimental and predicted values went down to 20%.

The better accuracy of the new models is even more evident in the estimation of the ultimate strain for which the MAPE values, though kept slightly high, went down below the 30% threshold. By comparing the accuracy between *Proposal 1* and *Proposal 2*, significant differences were not found, so *Proposal 1* can be successfully used for all the FRCM systems.

Overall, the values of the peak and ultimate strain estimated through the proposed models, together with the peak strength values obtained by using the proposals published in [14], may be successfully implemented in a numerical procedure capable of identifying a proper stress–strain law like the red curve in Figure 1, composed of two non-linear branches: the first one up to ($\varepsilon_{mc,\;}f_{mc})$ and characterized by an initial slope equal to the elastic modulus of the unconfined masonry, and the second (softening) one from the peak stress up to ($\varepsilon_{mcu,\;}80\%f_{mc})$. Similar procedures can be found in the literature [18].

Even though the present paper represents only a first contribution to the prediction of the compression strain at the peak and ultimate, and further research is needed on the topic, the performed study highlighted the improved accuracy of the proposed models with respect to the existing formulations. However, a better investigation of the effect of inorganic matrix is recommended, since the models currently proposed in the literature or guidelines deserve some criticisms that need to be addressed.

## Figures and Tables

**Table 1:** (**a**) Predictions of the peak axial strain $\varepsilon_{mc}$: existing models. (**b**) Predictions of the ultimate axial strain $\varepsilon_{mcu}$: existing models.

Ref.	ID	Model	*k_H_*	*k_V_*	$\rho_{f}$	$k_{\varepsilon }$
**(a)**						
Krevaikas [22]	*1A*	$\varepsilon_{mc}=\varepsilon_{m}+0.0139\cdot {\bar{f}}_{l,eff}$	$\left\{\begin{array}{cc} 1 & \left(CS\right)\\ 1-\frac{\left[{\left(H-2r_{c}\right)}^{2}+{\left(B-2r_{c}\right)}^{2}\right]}{3\cdot \left(B\cdot H\right)} & \left(RS\right) \end{array}\right.$	1( F−W)1−p′f2·D21−p′f2·minB,HDIS−W(CS)(RS)	$\left\{\begin{array}{ll} 4\frac{t_{f}}{D} & \left(CS\right)\\ 2t_{f}\frac{B+H}{B\cdot H} & \left(RS\right) \end{array}\right.$	0.85
	*1B*	$\varepsilon_{mc}=\varepsilon_{m}+0.018\cdot {{\bar{f}}_{l,eff}}^{1.84}$				
Koutas and Bournas [21]	*1C*	$\varepsilon_{mc}=\varepsilon_{m}+{0.043\cdot {\bar{f}}_{l,eff}}^{n}$ $n=\left\{\begin{array}{c} 1.00\;for\;C-FRCM\\ 0.95\;for\;G-FRCM\\ 0.75\;for\;B-FRCM \end{array}\right.$	$\left\{\begin{array}{cc} 1 & \left(CS\right)\\ 1-\frac{\left[{\left(H-2r_{c}\right)}^{2}+{\left(B-2r_{c}\right)}^{2}\right]}{3\cdot \left(B\cdot H\right)} & \left(RS\right) \end{array}\right.$	1( F−W)1−p′f2·D21−p′f2·minB,HDIS−W(CS)(RS)	$\left\{\begin{array}{ll} 4\frac{t_{f}}{D} & \left(CS\right)\\ 2t_{f}\frac{B+H}{B\cdot H} & \left(RS\right) \end{array}\right.$	0.85
Micelli et al. [18]	*1D*	$\varepsilon_{mc}=\varepsilon_{m}+{k_{mat}\cdot {\bar{f}}_{l,eff}}^{3.0}$ $k_{mat}=0.7\cdot {\left(\rho_{mat}\cdot \frac{f_{mat,c}}{f_{m}}\right)}^{1.2}$ $\rho_{mat}=\left\{\begin{array}{ll} \frac{4\cdot t_{mat}}{D} & \left(CS\right)\\ \frac{4\cdot t_{mat}}{\sqrt{B^{2}{+H}^{2}}} & \left(RS\right) \end{array}\right.$	$\left\{\begin{array}{cc} 1 & \left(CS\right)\\ 1-\frac{\left[{\left(H-2r_{c}\right)}^{2}+{\left(B-2r_{c}\right)}^{2}\right]}{3\cdot \left(B\cdot H\right)} & \left(RS\right) \end{array}\right.$	1( F−W)1−p′f2·D21−p′f2·minB,HDIS−W(CS)(RS)	$\left\{\begin{array}{ll} 4\frac{t_{f}}{D} & \left(CS\right)\\ 4\frac{t_{f}}{\sqrt{B^{2}{+H}^{2}}} & \left(RS\right) \end{array}\right.$	1.00
ACI 549.6R [5] *European approach*	*1E*	$\varepsilon_{mc}=\varepsilon_{m}\left[1+{k^{\prime }\cdot {\bar{f}}_{l,eff}}^{2}\right]$ $k^{\prime }=0.7{\left(\rho_{mat}\cdot \frac{f_{mat,c}}{f_{m}}\right)}^{2.2}$ $\rho_{mat}=\left\{\begin{array}{ll} \frac{4\cdot t_{mat}}{D} & \left(CS\right)\\ \frac{4\cdot t_{mat}}{\sqrt{B^{2}{+H}^{2}}} & \left(RS\right) \end{array}\right.$	$\left\{\begin{array}{cc} 1 & \left(CS\right)\\ {\left(\frac{B}{H}\right)}^{2}\cdot \left[1-\frac{\left[\left(\frac{B}{H}\right){\left(H-2r_{c}\right)}^{2}+{\left(\frac{H}{B}\right)\left(B-2r_{c}\right)}^{2}\right]}{3\cdot \left(B\cdot H\right)}\right] & \left(RS\right) \end{array}\right.$	1( F−W)1−p′f2·D21−p′f2·minB,HDIS−W(CS)(RS)	$\left\{\begin{array}{ll} 4\frac{t_{f}}{D} & \left(CS\right)\\ 4\frac{t_{f}}{\sqrt{B^{2}{+H}^{2}}} & \left(RS\right) \end{array}\right.$	1.00
**(b)**						
ACI 549.6R [5] *ACI approach*	*2A*	$\varepsilon_{mcu}=\varepsilon_{m}\left[1.5+12\cdot {\bar{f}}_{l,eff}{\cdot \left(\frac{\varepsilon_{f,u}}{\varepsilon_{m}}\right)}^{0.45}\right]$	$\left\{\begin{array}{cc} 1 & \left(CS\right)\\ {\left(\frac{B}{H}\right)}^{2}\cdot \left[1-\frac{\left[\left(\frac{B}{H}\right){\left(H-2r_{c}\right)}^{2}+{\left(\frac{H}{B}\right)\left(B-2r_{c}\right)}^{2}\right]}{3\cdot \left(B\cdot H\right)}\right] & \left(RS\right) \end{array}\right.$	1( F−W)1−p′f2·D21−p′f2·minB,HDIS−W(CS)(RS)	$\left\{\begin{array}{ll} 4\frac{t_{f}}{D} & \left(CS\right)\\ 4\frac{t_{f}}{\sqrt{B^{2}{+H}^{2}}} & \left(RS\right) \end{array}\right.$	1.00
Micelli et al. [18]	*2B*	$\begin{array}{c} \varepsilon_{mcu}=\varepsilon_{m}\left[1.36+{k^{\prime }\cdot {\bar{f}}_{l,eff}}^{2.0}\right]\\ k^{\prime }=0.01\cdot {\left(\frac{\varepsilon_{f,u}}{\varepsilon_{m}}\right)}^{0.5} \end{array}$	$\left\{\begin{array}{cc} 1 & \left(CS\right)\\ 1-\frac{\left[{\left(H-2r_{c}\right)}^{2}+{\left(B-2r_{c}\right)}^{2}\right]}{3\cdot \left(B\cdot H\right)} & \left(RS\right) \end{array}\right.$	1( F−W)1−p′f2·D21−p′f2·minB,HDIS−W(CS)(RS)	$\left\{\begin{array}{ll} 4\frac{t_{f}}{D} & \left(CS\right)\\ 4\frac{t_{f}}{\sqrt{B^{2}{+H}^{2}}} & \left(RS\right) \end{array}\right.$	1.00

**Table 2:** Distributions of specimens and datasets per FRCM system according to the following parameters: peak strain $\varepsilon_{mc}$ and ultimate strain $\varepsilon_{mcu}$.

Strain Data	ALL		B-FRCM		C-FRCM		G-FRCM		PBO-FRCM		S-FRCM	
	N. Specimens	n. Datasets	N. Specimens	n. Datasets	N. Specimens	n. Datasets	N. Specimens	n. Datasets	N. Specimens	n. Datasets	N. Specimens	n. Datasets
$\varepsilon_{mc}$	243	112	41	21	62	29	59	28	17	10	6	24
$\varepsilon_{mcu}$	160	68	40	20	19	11	53	22	–	–	48	15

**Table 3:** Dataset distributions per masonry type: AM = artificial masonry; NM = natural masonry.

Strain Data	ALL		B-FRCM		C-FRCM		G-FRCM		PBO-FRCM		S-FRCM	
	AM	NM	AM	NM	AM	NM	AM	NM	AM	NM	AM	NM
$\varepsilon_{mc}$	88	24	18	3	29	–	13	15	10	–	18	6
$\varepsilon_{mcu}$	49	19	17	3	11	–	7	15	–	–	14	1

**Table 4:** Predictions of the peak axial strain $\varepsilon_{mc}$ according to (a) *Model 1A–1C* (b) *Models 1D and 1E*: statistical analysis.

(a)																
FRCM	n	*Model 1A*					*Model 1B*					*Model 1C*				
		${∆}_{\delta }$	$\delta_{m}$	$\sigma_{\delta }$	${∆}_{\delta }$	$\delta_{m}$	${∆}_{\delta }$	$\delta_{m}$	$\sigma_{\delta }$	$\gamma_{\delta }$	$\delta_{n}$	${∆}_{\delta }$	$\delta_{m}$	$\sigma_{\delta }$	$\gamma_{\delta }$	$\delta_{n}$
		[-]	[-]	[-]	[-]	[-]	[-]	[-]	[-]	[-]	[%]	[-]	[-]	[-]	[-]	[%]
**ALL**	101	1.34	1.43	0.63	1.30	1.30	1.59	1.74	0.84	1.97	0.465	-	-	-	-	-
**B-FRCM**	21	1.37	1.74	0.95	1.57	1.83	1.61	2.09	1.22	1.80	0.087	0.79	0.92	0.48	0.88	0.144
**C-FRCM**	28	1.47	1.52	0.42	1.13	1.09	1.76	1.79	0.62	-0.17	0.310	0.97	0.95	0.34	0.03	0.531
**G-FRCM**	27	1.13	1.23	0.47	1.27	1.28	1.36	1.52	0.63	3.15	0.020	0.75	0.80	0.34	1.10	0.071
**PBO-FRCM**	6	1.94	2.00	0.47	1.83	1.52	2.55	2.69	0.81	0.99	0.026	-	-	-	-	-
**S-FRCM**	19	0.98	1.05	0.35	0.87	0.98	1.26	1.29	0.42	-0.21	0.022	-	-	-	-	-
**(b)**																
**FRCM**	**n**	***Model 1D***					***Model 1E***									
		${∆}_{\delta }$	$\delta_{m}$	${∆}_{\delta }$	$\delta_{m}$	${∆}_{\delta }$	$\delta_{m}$	${∆}_{\delta }$	$\delta_{m}$	${∆}_{\delta }$	$\delta_{m}$					
		**[-]**	**[-]**	**[-]**	**[-]**	**[-]**	**[-]**	**[-]**	**[-]**	**[-]**	**[-]**					
**ALL**	101	1.30	1.30	1.30	1.30	1.30	1.30	1.30	1.30	1.30	1.30					
**B-FRCM**	21	1.57	1.83	1.57	1.83	1.57	1.83	1.57	1.83	1.57	1.83					
**C-FRCM**	28	1.13	1.09	1.13	1.09	1.13	1.09	1.13	1.09	1.13	1.09					
**G-FRCM**	27	1.27	1.28	1.27	1.28	1.27	1.28	1.27	1.28	1.27	1.28					
**PBO-FRCM**	6	1.83	1.52	1.83	1.52	1.83	1.52	1.83	1.52	1.83	1.52					
**S-FRCM**	19	0.87	0.98	0.87	0.98	0.87	0.98	0.87	0.98	0.87	0.98					

**Table 5:** Predictions of the ultimate axial strain $\varepsilon_{mcu}$ according to *Model 2B*: statistical analysis.

FRCM	n	*Model 2B*				
		${∆}_{\delta }$	$\delta_{m}$	$\sigma_{\delta }$	$\gamma_{\delta }$	$\delta_{n}$
		[-]	[-]	[-]	[-]	[%]
**ALL**	68	1.74	2.33	1.53	2.51	0.718
**B-FRCM**	20	1.74	2.45	1.64	1.89	0.184
**C-FRCM**	11	2.76	2.66	1.32	0.29	0.140
**G-FRCM**	22	1.65	1.97	0.81	0.68	0.094
**PBO-FRCM**	–	–	–	–	–	–
**S-FRCM**	15	1.66	2.48	2.23	2.82	0.300

**Table 6:** Peak strain $\varepsilon_{mc}$ predictions: new proposal suitable for any FRCM system (*Proposal 1*).

FRCM	n	$\alpha_{1}$	$\alpha_{2}$	Proposal 1	${∆}_{\delta }$	$\delta_{m}$	$\sigma_{\delta }$	$\gamma_{\delta }$	$\delta_{n}$	${\left(E_{rr}\right)}_{m}$
		[-]	[-]	[-]	[-]	[-]	[-]	[-]	[%]	[%]
**ALL**	101	0.006	0.37	$\varepsilon_{mc}=\varepsilon_{m}+0.006\cdot {{\bar{f}}_{l,eff}}^{0.37}$	1.19	1.28	0.52	1.60	0.275	27
**B-FRCM**	21				1.19	1.40	0.67	1.40	0.060	29
**C-FRCM**	28				1.47	1.58	0.52	1.64	0.182	32
**G-FRCM**	27				0.94	0.99	0.23	0.61	0.009	20
**PBO-FRCM**	6				1.57	1.62	0.35	1.04	0.011	36
**S-FRCM**	19				1.00	1.02	0.30	0.59	0.012	24

**Table 7:** Peak strain $\varepsilon_{mc}$ predictions: new proposal specified for each FRCM system (*Proposal 2*).

FRCM	n	$\alpha_{1}$	$\alpha_{2}$	Proposal 2	${∆}_{\delta }$	$\delta_{m}$	$\sigma_{\delta }$	$\gamma_{\delta }$	$\delta_{n}$	${\left(E_{rr}\right)}_{m}$
		[-]	[-]	[-]	[-]	[-]	[-]	[-]	[%]	[%]
**B-FRCM**	21	0.010	0.53	$\varepsilon_{mc}=\varepsilon_{m}+0.010\cdot {{\bar{f}}_{l,eff}}^{0.53}$	21	1.07	1.38	0.67	1.47	28
**C-FRCM**	28	0.015	0.46	$\varepsilon_{mc}=\varepsilon_{m}+0.015\cdot {{\bar{f}}_{l,eff}}^{0.46}$	28	1.07	1.12	0.30	1.25	19
**G-FRCM**	27	0.002	0.13	$\varepsilon_{mc}=\varepsilon_{m}+0.004\cdot {{\bar{f}}_{l,eff}}^{0.13}$	27	1.01	1.13	0.25	0.45	17
**PBO-FRCM**	6	0.007	0.11	$\varepsilon_{mc}=\varepsilon_{m}+0.007\cdot {{\bar{f}}_{l,eff}}^{0.11}$	6	1.06	1.12	0.24	1.13	13
**S-FRCM**	19	0.006	0.35	$\varepsilon_{mc}=\varepsilon_{m}+0.006\cdot {{\bar{f}}_{l,eff}}^{0.35}$	19	1.00	1.02	0.30	0.71	24

**Table 8:** Ultimate strain $\varepsilon_{mcu}$ predictions: new proposal suitable for any FRCM system (*Proposal 1*).

FRCM	n	$\alpha_{1}$	$\alpha_{2}$	$\alpha_{3}$	Proposal 1	${∆}_{\delta }$	$\delta_{m}$	$\sigma_{\delta }$	$\gamma_{\delta }$	$\delta_{n}$	${\left(E_{rr}\right)}_{m}$
		[-]	[-]	[-]	[-]	[-]	[-]	[-]	[-]	[%]	[%]
**ALL**	68	0.64	0.53	1.00	$\varepsilon_{mcu}=\varepsilon_{m}\left[1.36+0.64\cdot \left(\frac{\varepsilon_{f,u}}{\varepsilon_{m}}\right)\cdot {{\bar{f}}_{l,eff}}^{0.53}\right]$	1.07	1.24	0.66	3.06	0.369	25
**B-FRCM**	20					1.14	1.49	0.96	2.52	0.094	22
**C-FRCM**	11					1.08	1.10	0.25	0.49	0.018	19
**G-FRCM**	22					0.97	1.09	0.38	0.67	0.033	28
**PBO-FRCM**	-					-	-	-	-	-	-
**S-FRCM**	15					1.06	1.24	0.63	1.80	0.223	29

**Table 9:** Ultimate strain $\varepsilon_{mcu}$ predictions: new proposal specified for each FRCM system (*Proposal 2)*.

FRCM	n	$\alpha_{1}$	$\alpha_{2}$	$\alpha_{3}$	Proposal 2	${∆}_{\delta }$	$\delta_{m}$	$\sigma_{\delta }$	$\gamma_{\delta }$	$\delta_{n}$	${\left(E_{rr}\right)}_{m}$
		[-]	[-]	[-]	[-]	[-]	[-]	[-]	[-]	[%]	[%]
**B-FRCM**	20	1.00	0.66	1.00	$\varepsilon_{mcu}=\varepsilon_{m}\left[1.36+\left(\frac{\varepsilon_{f,u}}{\varepsilon_{m}}\right)\cdot {{\bar{f}}_{l,eff}}^{0.66}\right]$	1.05	1.41	0.94	2.60	0.084	22
**C-FRCM**	11	1.29	0.66	0.72	$\varepsilon_{mcu}=\varepsilon_{m}\left[1.36+1.29\cdot {\left(\frac{\varepsilon_{f,u}}{\varepsilon_{m}}\right)}^{0.72}\cdot {{\bar{f}}_{l,eff}}^{0.66}\right]$	1.00	1.03	0.23	0.30	0.019	17
**G-FRCM**	22	0.65	0.58	1.00	$\varepsilon_{mcu}=\varepsilon_{m}\left[1.36+0.65\cdot \left(\frac{\varepsilon_{f,u}}{\varepsilon_{m}}\right)\cdot {{\bar{f}}_{l,eff}}^{0.58}\right]$	0.99	1.13	0.40	0.64	0.035	27
**PBO-FRCM**	-	-	-	-	-	-	-	-	-	-	-
**S-FRCM**	15	0.65	0.45	1.00	$\varepsilon_{mcu}=\varepsilon_{m}\left[1.36+0.65\cdot \left(\frac{\varepsilon_{f,u}}{\varepsilon_{m}}\right)\cdot {{\bar{f}}_{l,eff}}^{0.45}\right]$	1.01	1.17	0.60	1.86	0.219	29

## Data Availability

The data presented in this study are available upon request from the corresponding author due to ethical reasons.

## Appendix A. Database Collection

The main details of 112 datasets collected from the literature are provided in Table A1–A5 for specimens confined with B-, C-, G-, PBO-, and S- systems, respectively. In detail, the following information is reported for each dataset:

- the reference to the manuscript (“source”) from which the experimental data were obtained;
- the nature of the masonry (*type*) and its mass density $\left(g_{m}\right)$;
- the label of each single dataset (ID);
- the number of specimens per dataset (N);
- the shape of the column cross-section (R = rectangular, S = square, C = circular);
- the width (B), depth (H), or diameter (D) of the cross-section and the height (H) of the column;
- the corner radius ($r_{c}$) of the column cross-section in the cases of S and R specimens;
- the compressive strength of the unconfined masonry ($f_{m}$), corresponding axial strain $\left(\varepsilon_{m}\right)$, and ultimate one $\left(\varepsilon_{mu}\right)$;
- the elastic modulus in compression ($E_{mat})$, compressive strength ($f_{mat,c}$), and bending strength ($f_{mat,b}$) of the inorganic matrix;
- the overall thickness of the FRCM jacket ($t_{mat}$);
- the density $(\gamma_{f}),$ tensile strength $\left(f_{f,u}\right)$, elastic modulus $\left(E_{f}\right)$, and ultimate strain of the dry strengthening sheet ($\varepsilon_{f,u})$ and the equivalent thickness of the single layer ($t_{f,j})$;
- the number $\left(n_{f}\right)$ and the overlapping length $\left(L_{b}\right)$ of the FRCM layers;
- the confinement layout (F-W = full-wrapping, DIS-W = discontinuous wrapping);
- the ratio between the compressive strength of the confined masonry $\left(f_{mc}\right)$ and the strength of the unconfined masonry $\left(f_{m}\right)$;
- the ratio between the compressive strain at the peak strength of the confined masonry $\left(\varepsilon_{mc}\right)$ and the corresponding peak strain of the unconfined masonry $\left(\varepsilon_{m}\right)$;
- the ratio between the conventional ultimate strain—at 15–20% strength decay in the softening branch—($\varepsilon_{mcu})$ and $\varepsilon_{m}$;
- the dominant failure mode (FM) exhibited by test specimens (i.e., JF = jacket failure, DB = debonding of the reinforcement at the overlap region, S = fiber–matrix slippage (S)).
- Concerning the masonry mass density, it is noted that when it was not found in the literature, a typical average value of $g_{m}$ for that type of masonry was assumed and is indicated in the tables (in these cases, the $g_{m}$ values are reported in italics).

**Table A1:** Experimental datasets concerning B-FRCM-confined specimens (21 datasets, 41 specimens).

Source	Masonry		Specimens										Strengthening												Results			
													Matrix’s Properties				Fabric Mesh’s Properties					Jacket						
	*Type*	*g_m_* ^1^	*Dataset ID*	*N*	*Shape*	*B*	*H*	*L*	*r_c_*	*f_m_*	*ε_m_*	*ε_m,u_*	*f_mat,c_*	*f_mat,b_*	*E_mat_*	*t_mat_*	*γ_f_*	*f_f,u_*	*E_f_*	*ε_f,u_*	*t_f,j_*	*n_f_*	*L_b_*	*Layout*	*f_mc_*/*f_m_*	*ε_mc_*/*ε_m_*	*ε_mcu_*/*ε_m_*	FM
		[kg/m^3^]		[-]	[-]	[mm]	[mm]	[mm]	[mm]	[MPa]	[-]	[-]	[MPa]	[MPa]	[GPa]	[mm]	[g/m^2^]	[MPa]	[GPa]	[%]	[mm]	[-]	[mm]	[-]	[MPa]	[-]	[-]	
[23]	CB	*1700*	M1_BF_1,2	2	S	230	230	960	20	4.68	0.01420	0.01680	0.55	0.44	-	10	250	1542	89.00	1.80	0.039	1	460	F-W	1.74	1.50	1.75	JF
			M3_BF_1,2	2	S					9.42	0.01240	0.01390	4.54	3.13	-	10						1	460	F-W	1.14	1.16	1.29	JF
[24]	CB	*1700*	S-2B-L (1), (2)	2	S	360	360	900	12	1.61	0.01020	0.01180	0.87	-	-	18	170	-	370.00	1.62	0.046	2	360	F-W	1.10	1.76	2.43	JF
			S-2B-F (1), (2)	2	S								9.83	-	-	18						2	360	F-W	1.04	2.70	3.24	JF
			R-2B-L (1), (2)	2	R	360	630	900	12	1.45	0.01495	0.01830	0.87	-	-	18						2	360	F-W	0.99	1.47	1.90	JF
			R-2B-F (1), (2)	2	R								9.83	-	-	18						2	360	F-W	1.00	1.13	1.57	JF
[25]	LS	1600	S_Bgrid	1	S	400	400	2100	20	4.78	0.00040	0.00050	13.61	15.54	-	15	250	1602	89.00	1.80	0.039	1	100	F-W	1.68	3.25	4.75	DB
[26]	CB	*1700*	C-B-1	1	S	250	250	770	20	5.35	0.00260	-	12.85	2.65	-	6	-	930	70.30	1.40	0.064	1	-	F-W	1.44	3.77	7.31	DB-JF
			C-B-2	1	S											9						2	-	F-W	1.29	6.08	10.00	DB-JF
[21]	CB	*1700*	A_B3	1	S	215	215	670	20	9.97	0.00370	0.00510	37.80	9.30	-	20 ^a^	220	-	89.00	-	0.037	3	195	F-W	1.15	1.89	2.38	JF
			A_B5	1	S											30 ^a^						5	195	F-W	1.31	3.38	3.57	JF
			A_B7	1	S											40 ^a^						7	195	F-W	1.44	4.11	4.35	JF
			B_B3	1	R	215	440	670	20	8.79	0.00770	0.01350				20 ^a^						3	195	F-W	1.03	1.42	1.95	JF
			B_B5	1	R											30 ^a^						5	195	F-W	1.11	2.13	2.34	JF
			B_B7	1	R											40 ^a^						7	195	F-W	1.17	2.35	-	JF
[27]	LS	2000	BG.01.01, 02, 03, 04	4	C	83		218	-	20.79	0.00264	-	28.00	10.00	11.00	7	250	1542	89.00	1.80	0.039	1	130	F-W	1.21	1.47	2.28	-
			BG.03.01, 02, 03, 04	4	C											10						3	130	F-W	1.24	1.44	6.38	JF
[28]	CB	*1700*	C1, 2, 3, 4M_W1L	4	C	94		190	-	25.19	0.00360	0.00380	25.00	8.00	10.00	4	250	1542	89.00	1.80	0.039	1	100	F-W	1.27	1.60	2.06	JF
			C1, 2, 3M_W2L	3	C											6						2	100	F-W	1.38	1.74	2.36	JF
			C1, 2, 3M_C1L	3	C					19.85	0.00340	0.00380				4						1	100	F-W	1.66	1.46	2.22	JF
			C1, 2M_C2L	2	C											6						2	100	F-W	1.85	1.76	2.49	JF

^a^ assumed value. ^1^ the assumed values are in italics.

**Table A2:** Experimental datasets concerning C-FRCM-confined specimens (29 datasets, 62 specimens).

Source	Masonry		Specimens										Strengthening												Results			
													Matrix’s Properties				Fabric Mesh’s Properties					Jacket						
	*Type*	*g*_m_^1^	*Dataset ID*	*N*	*Shape*	*B*	*H*	*L*	*r_c_*	f_m_	*ε_m_*	*ε_mu_*	*f_mat,c_*	*f_mat,b_*	*E_mat_*	*t_mat_*	*γ_f_*	*f_f,u_*	*E_f_*	*ε_f,u_*	*t_f,j_*	*n_f_*	*L_b_*	*Layout*	*f_mc_*/*f_m_*	*ε_mc_*/*ε_m_*	*ε_mcu_*/*ε_m_*	FM
		[kg/m^3^]		[-]	[-]	[mm]	[mm]	[mm]	[mm]	[MPa]	[-]	[-]	[MPa]	[MPa]	[GPa]	[mm]	[g/m^2^]	[MPa]	[GPa]	[%]	[mm]	[-]	[mm]	[-]	[MPa]	[-]	[-]	
[8]	CB	*1700*	C1_1_R20	3	S	240	240	300	20	1.19	0.00390	0.01350	5.27	-	-	5	220	4800	220.00	2.20	0.047	1	100	F-W	1.74	4.10	5.09	JF
			C1_1_R10	3	S	240	240	300	10													1	100	F-W	2.86	3.50	6.02	JF
			C2_1_R10	3	S											10						2	100	F-W	3.87	4.74	6.62	JF
			C3_1_R10	3	S											15						3	100	F-W	5.07	3.59	4.69	JF
			C1_1_R10	3	S	240	240	310	10	4.07	0.00495	-	35.00	-	-	6	270	3800	230.00	1.65	0.075	1	120	F-W	1.05	3.39	-	DB-JF
			C2_1_R10	3	S											9						2	120	F-W	1.28	4.04	-	DB-JF
			C3_1_R10	3	S											12						3	120	F-W	1.57	4.31	-	DB-JF
			C1_1_R20	3	S	240	240	310	20							6						1	120	F-W	0.87	3.37	-	DB-JF
			C2_1_R20	3	S											9						2	120	F-W	1.89	3.89	-	DB-JF
			C3_1_R20	3	S											12						3	120	F-W	2.22	7.27	-	DB-JF
			C1_1.5_R10	3	R	240	360	310	10	4.76	0.02640	-				6						1	180	F-W	1.08	*	-	DB-JF
			C2_1.5_R10	3	R											9						2	180	F-W	1.28	1.20	-	DB-JF
			C3_1.5_R10	3	R											12						3	180	F-W	1.55	1.44	-	DB-JF
			C1_2_R10	3	R	240	480	310	10	7.38	0.01300	-				6						1	240	F-W	0.96	1.54	-	DB-JF
			C2_2_R10	3	R											9						2	240	F-W	1.18	2.32	-	DB-JF
			C3_2_R10	3	R											12						3	240	F-W	1.23	2.08	-	DB-JF
[9,29]	CB	1700	CP_RC170_01,02	2	S	380	380	1000	20	12.63	0.00472		6.52	-	5.90	7	170	2363	220.36	1.80	0.047	2	250	F-W	1.04	1.43	-	JF
[21]	CB	*1700*	A_CH1	1	S	215	215	670	20	9.97	0.00370	0.00500	37.80	9.30	-	10 ^a^	348	4800	225.00	2.13	0.095	1	195	F-W	1.13	2.05	2.36	JF
			A_CH2	1	S	215	215	670	20				37.80	9.30	-	15 ^a^	348	4800	225.00	2.13	0.095	2	195	F-W	1.47	2.84	3.76	JF
			A_CH3	1	S											20 ^a^						3	195	F-W	1.61	4.08	4.24	JF
			B_CH1	1	R	215	440	670	20	8.79	0.00770	0.00139				10 ^a^						1	195	F-W	1.18	1.62	2.38	JF
			B_CH2	1	R											15 ^a^						2	195	F-W	1.35	1.65	-	JF
			B_CH3	1	R											20 ^a^						3	195	F-W	1.37	1.83	-	JF
			A_CL1	1	S	215	215	670	20	9.97	0.00370	0.00500				10 ^a^	220	3800	225.00	1.69	0.062	1	195	F-W	1.07	1.89	1.98	JF
			A_CL2	1	S											15 ^a^						2	195	F-W	1.40	3.03	-	JF
			A_CL3	1	S											20 ^a^						3	195	F-W	1.45	3.43	-	JF
			B_CL1	1	R	215	440	670	20	8.79	0.00770	0.00139				10 ^a^						1	195	F-W	1.09	1.05	1.25	JF
			B_CL2	1	R											15 ^a^						2	195	F-W	1.19	1.29	1.76	JF
			B_CL3	1	R											20 ^a^						3	195	F-W	1.52	1.73	-	JF

** ε_mc,u_/ε_m,u_* < 1. ^a^ assumed value. ^1^ the assumed values are in italics.

**Table A3:** Experimental datasets concerning G-FRCM-confined specimens (28 datasets, 59 specimens).

Source	Masonry		Specimens										Strengthening												Results			
													Matrix’s Properties				Fabric Mesh’s Properties					Jacket						
	*Type*	*g_m_* ^1^	*Dataset ID*	*N*	*Shape*	*B*	*H*	*L*	*r_c_*	*f_m_*	*ε_m_*	*ε_mu_*	*f_mat,c_*	*f_mat,b_*	*E_mat_*	*t_mat_*	*γ_f_*	*f_f,u_*	*E_f_*	*ε_f,u_*	*t_f,j_*	*n_f_*	*L_b_*	*Layout*	*f_mc_*/*f_m_*	*ε_mc_*/*ε_m_*	*ε_mcu_*/*ε_m_*	FM
		[kg/m^3^]		[-]	[-]	[mm]	[mm]	[mm]	[mm]	[MPa]	[-]	[-]	[MPa]	[MPa]	[GPa]	[mm]	[g/m^2^]	[MPa]	[GPa]	[%]	[mm]	[-]	[mm]	[-]	[MPa]	[-]	[-]	
[25]	LS	1600	S_Grid	1	S	400	400	2100	20	4.78	0.00040	0.00050	13.61	5.54	-	15	225	1296	72.00	1.80	0.035	1	100	F-W	1.50	4.25	5.25	DB
[29]	CB	1700	CP_RV320_01, 02	2	S	380	380	1000	20	12.63	0.00472	-	6.52	-	5.90	7	300	1200	60.00	2.00	0.064	2	250	F-W	1.07	1.30	-	JF
[30]	LS	1530	FRCM_M4_1, 2, 3	3	S	250	250	500	30	7.61	0.00520	0.01800	4.15	-	-	10	120	742.4	37.12	2.00	0.250	1	250	F-W	1.06	1.03	1.86	DB-JF-S
			FRCM_M7_1, 2, 3	3	S								7.26	-	-	10						1	250	F-W	1.31	1.32	2.24	JF
			FRCM_M23_1, 2, 3	3	S	250	250	500	30				22.93	-	-	10						1	250	F-W	1.87	1.07	1.47	JF
[31]	LS	1800	CM8-1, 2, 3	3	S	250	250	500	20	5.23	0.00450	0.00962	1.67	0.79	-	10	220	1400	70.00	2.00	0.088	1	250	F-W	1.17	2.17	3.55	JF
			CM13-1, 2, 3	3	S								2.16	0.95	-	10						1	250	F-W	1.11	1.27	3.57	JF
			CC25-1, 2, 3	3	S								16.84	4.78	-	10						1	250	F-W	1.14	1.31	2.80	JF
[21]	CB	*1700*	A_G3	1	S	215	215	670	20	9.97	0.00370	0.00500	37.80	9.30	-	20 ^a^	220	1400	74.00	1.89	0.044	3	195	F-W	1.28	1.43	1.59	JF
			A_G5	1	S											30 ^a^						5	195	F-W	1.35	2.03	-	JF
			A_G7	1	S											40 ^a^						7	195	F-W	1.49	2.14	-	JF
			B_G3	1	R	215	440	670	20	8.79	0.00770	0.00139				20 ^a^						3	195	F-W	1.02	1.61	-	JF
			B_G5	1	R											30 ^a^						5	195	F-W	1.08	1.86	-	JF
			B_G7	1	R											40 ^a^						7	195	F-W	1.12	1.23	-	JF
[32]	TU	1400	3, 4_RM—UniSal	2	S	250	250	615	20	2.25	0.00505	0.00635	9.10	-	-	10	300	1929	108.00	1.80	0.060	1	250	F-W	1.10	1.99	3.80	JF
			5, 6_RM—UniSal	2	S											15						2	250	F-W	1.66	2.16	3.91	JF
			7, 8_RM—UniSal	2	S											20						3	250	F-W	2.08	1.71	2.84	JF
	TU	1400	3, 4_RM—UniPa	2	S	250	250	615	20	2.85	0.00355	0.00455	9.10	-	-	10	300	1929	108.00	1.80	0.060	1	250	F-W	1.00	1.89	4.25	JF
			5, 6_RM—UniPa	2	S											15						2	250	F-W	1.10	1.87	3.62	JF
			7, 8_RM—UniPa	2	S											20						3	250	F-W	1.52	1.42	3.68	JF
	CB	1600	3, 4_RM—UniCal	2	S	250	250	575	20	3.58	0.00410	0.00540	9.10	-	-	10	300	1929	108.00	1.80	0.060	1	250	F-W	1.59	1.43	1.79	JF
			5, 6_RM—UniCal	2	S											15						2	250	F-W	2.27	1.85	1.79	JF
			7, 8_RM—UniCal	2	S											20						3	250	F-W	2.32	1.44	1.80	JF
	CB	1600	3, 4_RM—UniNa	2	S	250	250	575	20	5.62	0.00465	0.00615	9.10	-	-	10	300	1929	108.00	1.80	0.060	1	250	F-W	1.18	1.15	1.63	JF
			5, 6_RM—UniNa	2	S											15						2	250	F-W	1.05	1.16	1.41	JF
			7, 8_RM—UniNa	2	S											20						3	250	F-W	1.37	1.68	2.25	JF
[27]	LS	2000	GG.01.01, 02, 03, 04	4	C	81		218	-	20.79	0.00264	-	28.00	10.00	11.00	7	125	1276	72.00	1.80	0.024	1	130	F-W	1.01	*	1.66	JF
			GG.03.01, 02, 03, 04	4	C											10						3	130	F-W	1.26	1.37	2.21	JF

** ε_mc_ /ε_m_* < 1. ^a^ assumed value. ^1^ the assumed values are in italics.

**Table A4:** Experimental datasets concerning PBO-FRCM-confined specimens (10 datasets, 17 specimens).

Source	Masonry		Specimens										Strengthening												Results			
													Matrix’s Properties				Fabric Mesh’s Properties					Jacket						
	*Type*	*g_m_* ^1^	*Dataset ID*	*N*	*Shape*	*B*	*H*	*L*	*r_c_*	*f_m_*	*ε_m_*	*ε_mu_*	*f_mat,c_*	*f_mat,b_*	*E_mat_*	*t_mat_*	*γ_f_*	*f_f,u_*	*E_f_*	*ε_f,u_*	*t_f,j_*	*n_f_*	*L_b_*	*Layout*	*f_mc_*/*f_m_*	*ε_mc_*/*ε_m_*	*ε_mcu_*/*ε_m_*	FM
		[kg/m^3^]		[-]	[-]	[mm]	[mm]	[mm]	[mm]	[MPa]	[-]	[-]	[MPa]	[MPa]	[GPa]	[mm]	[g/m^2^]	[MPa]	[GPa]	[%]	[mm]	[-]	[mm]	[-]	[MPa]	[-]	[-]	
[10]	CB	*1700*	W_C-1, 2, 3	3	R	90	200	380	10	36.33	0.00557	-	28.40	-	-	8	-	5800	270.00	2.15	0.092	1	100	F-W	1.16	*	-	S
			SQ_C-1, 2, 3	3	S	102	100	240	10	29.07	0.00527	-										1	100	F-W	1.29	2.32	-	S
			RECT_1_C-1, 2, 3	3	R	102	152	240	10	22.00	0.00603	-										1	100	F-W	1.72	2.32	-	S
			RECT_2_C-1, 2	2	R	102	204	240	10	8.90	0.00393	-										1	100	F-W	3.97	1.59	-	S
[26]	CB	*1700*	C-P-1	1	S	250	250	770	20	5.35	0.00260	-	36.16	5.50	-	6	-	3400	211.40	2.50	0.046	1	-	F-W	1.98	10.12 **	-	JF
			C-P-2	1	S						0.00260	-				9						2	-	F-W	2.54	9.23 **	-	DB
			C-P-3	1	S						0.00260	-				12						3	-	F-W	2.85	10.73 **	-	DB
[33]	CB	*1700*	C-D-P-1L	1	S	250	250	770	20	5.19	0.00250	-	36.16	5.50	-	6	-	3400	211.40	2.59	0.046	1	-	DIS-W	1.34	3.08	-	DB
			C-D-P-2L	1	S						0.00250	-				9						2	-	DIS-W	1.43	3.24	-	DB
			C-D-P-3L	1	S						0.00250	-				12						3	-	DIS-W	1.93	5.12	-	DB

** ε_mc_/ε_m_* < 1. ** values excluded in the analysis. ^1^ the assumed values are in italics.

**Table A5:** Experimental datasets concerning S-FRCM-confined specimens (24 datasets, 64 specimens).

Source	Masonry		Specimens										Strengthening													Strengthening			
													Matrix’s Properties				Fabric Mesh’s Properties					Jacket							
	*Type*	*g_m_* ^1^	*Dataset ID*	*N*	*Shape*	*B*	*H*	*L*	*r_c_*	*f_m_*	*ε_m_*	*ε_mu_*	*f_mat,c_*	*f_mat,b_*	*E_mat_*	*t_mat_*	*γ_f_*	*f_f,u_*	*E_f_*	*ε_f,u_*	*t_f,j_*	*n_f_*	*L_b_*	*k_V_*	*k_H_*	*f_mc_*/*f_m_*	*ε_mc_*/*ε_m_*	*ε_mcu_*/*ε_m_*	FM
		[kg/m^3^]		[-]	[-]	[mm]	[mm]	[mm]	[mm]	[MPa]	[-]	[-]	[-]	[MPa]	[GPa]	[mm]	[g/m^2^]	[MPa]	[GPa]	[%]	[mm]	[-]	[mm]	[-]	[-]	[MPa]	[-]	[-]	
[34,35]	CB	*1700*	C-1-6-0-1, 2, 3, 4	4	S	250	250	720	0	7.38	0.00580	0.00857	15.00	5.00	9.00	8	670	2900	205.00	2.00	0.084	1	250	1.00	0.33	1.27	1.72	2.19	DB
			C-1-6-9-1, 2, 3	3	S	250	250	720	9.5		0.00580	0.00857									0.084	1	250	1.00	0.43	1.28	2.00	2.48	DB
			C-1-6-38-1, 2, 3, 4	4	S	250	250	720	38.1		0.00580	0.00857									0.084	1	250	1.00	0.68	1.41	1.84	2.39	DB
			C-1-12-9-1, 2, 3, 4	4	S	250	250	720	9.5		0.00580	0.00857					1200				0.169	1	250	1.00	0.43	1.35	1.31	1.58	DB
[35]	CB	*1700*	C-2-6-0-1, 2, 3, 4	4	S	250	250	720	0	7.38	0.00580	0.00857	47.10	4.40	-	10	670	2900	205.00	2.00	0.084	1	240	1.00	0.33	1.26	1.65	1.96	DB/JF
			C-2-6-9-1, 2, 3, 4	4	S	250	250	720	9.5		0.00580	0.00857									0.084	1	200	1.00	0.43	1.33	1.94	2.85	DB
			C-2-6-38-1, 2, 3, 4	4	S	250	250	720	38.1		0.00580	0.00857									0.084	1	200	1.00	0.68	1.39	1.94	3.42	DB/JF
			C-2-12-9-1, 2, 3, 4	4	S	250	250	720	9.5		0.00580	0.00857					1200				0.169	1	200	1.00	0.43	1.42	1.65	2.00	DB
[36]	CB	*1700*	C-12-1-1-0	1	S	250	250	770	20	5.19	0.00260	-	12.85	2.65	-	6	1200	2900	205.00	2.00	0.169	1	250	1.00	0.53	2.13	1.94	-	DB
			C-12-2-1-0	1	S						0.00260	-				9						2	250	1.00	0.53	1.96	2.78	-	DB
			C-12-3-1-0	1	S						0.00260	-				12						3	250	1.00	0.53	2.91	5.94	-	DB
[26]	CB	*1700*	C-S-1, 2	1	S	250	250	770	20	5.19	0.00250	-	12.85	2.65	-	6	1200	2900	205.00	2.00	0.169	1	250	1.00	0.53	1.82	2.64	-	DB
[31]	TU	1400	2, 3, 4_RM—UniSa	3	S	250	250	615	20	3.89	0.00540	0.00730	13.40	-	-	10	670	3080	193.40	2.17	0.084	1	375	1.00	0.53	1.28	1.19	-	DB
			5, 6_RM—UniSa	2	S						0.00540	0.00730				15						2	375	1.00	0.53	1.48	*	-	DB
			7, 8_RM—UniSa	2	S						0.00540	0.00730				20						3	375	1.00	0.53	1.60	*	-	DB
	TU	1400	2, 3, 4_RM—UniFi	3	S	250	250	615	20	4.33	0.00310	0.00360	13.40	-	-	10	670	3080	193.40	2.17	0.084	1	375	1.00	0.53	1.12	1.23	13.17	DB
			5, 6_RM—UniFi	2	S						0.00310	0.00360				15						2	375	1.00	0.53	1.23	13.10 **	-	DB
			7, 8_RM—UniFi	2	S						0.00310	0.00360				20						3	375	1.00	0.53	1.26	15.35 **	-	DB
	CB	1600	2, 3, 4_RM—UniBo	3	S	250	250	575	20	9.09	0.00440	0.00690	13.40	-	-	10	670	3080	193.40	2.17	0.084	1	375	1.00	0.53	1.15	1.33	2.26	DB
			5, 6_RM—UniBo	2	S						0.00440	0.00690				15						2	375	1.00	0.53	1.28	1.80	3.58	DB
			7, 8_RM—UniBo	2	S						0.00440	0.00690				20						3	375	1.00	0.53	1.38	1.78	7.18	DB
	CB	1600	2, 3, 4_RM—PoliMi	3	S	250	250	575	20	4.92	0.00800	-	13.40	-	-	10	670	3080	193.40	2.17	0.084	1	375	1.00	0.53	2.07	*	1.29	DB
			5, 6_RM—PoliMi	2	S						0.00800	-				15						2	375	1.00	0.53	2.24	1.11	2.10	DB
			7, 8_RM—PoliMi	1	S						0.00800	-				20						3	375	1.00	0.53	2.52	1.29	2.26	DB

** ε_mc_ /ε_m_* < 1. ** values excluded in the analysis. ^1^ the assumed value are in italics.
